# Dissociable Effects of Valence and Arousal on Different Subtypes of Old/New Effect: Evidence from Event-Related Potentials

**DOI:** 10.3389/fnhum.2015.00650

**Published:** 2015-12-11

**Authors:** Huifang Xu, Qin Zhang, Bingbing Li, Chunyan Guo

**Affiliations:** ^1^Department of Psychology, Capital Normal UniversityBeijing, China; ^2^Graduate School of Beijing University of Chinese MedicineBeijing, China

**Keywords:** valence, arousal, familiarity, recollection, post-retrieval processing, conceptual, sensory

## Abstract

Here, we utilized the study-test paradigm combined with recognition confidence assessment and behavioral and event-related potential (ERP) measurements to investigate the effects of valence and arousal on the different subtypes of the old-new effect. We also test the effect of valence and arousal at encoding stage to investigate the underlying mechanism of the effect of the two emotional dimension on different retrieval process. In order to test the effects of valence and arousal on old/new effect precisely, we used the “subject-oriented orthogonal design” which manipulated valence and arousal independently according to subjects’ verbal reporting to investigate the effects of valence and arousal on old/new effect respectively. Three subtypes of old/new effect were obtained in the test phase, which were FN400, LPC, and late positivity over right frontal. They are supposed to be associated with familiarity, recollection, and post-retrieval processes respectively according to previous studies. For the FN400 component, valence affected mid-frontal negativity from 350–500 ms. Pleasant items evoked an enhanced ERP old/new effect relative to unpleasant items. However, arousal only affected LPC amplitude from 500–800 ms. The old/new effect for high-arousal items was greater than for low-arousal items. Valence also affected the amplitude of a positive-going slow wave at right frontal sites from 800–1000 ms, possibly serving as an index of post-retrieval processing. At encoding stage, the valence and arousal also have dissociable effect on the frontal slow wave between 350–800 ms and the centro-parietal positivity in 500–800 ms. The pleasant items evoked a more positive frontal slow wave relative to unpleasant ones, and the high arousal items evoked a larger centro-parietal positivity relative to low arousal ones. These results suggest that valence and arousal may differentially impact these different memory processes: valence affects familiarity and post-retrieval processing, whereas arousal affects recollection. These effects may be due to the conceptual encoding strategies for pleasant information and sensory encoding strategies for high arousal information.

## Introduction

Compared to non-emotional events, people tend to remember emotional events with more perceptual and sensory detail, recall them with greater accuracy, and have greater confidence in the accuracy of the recalled details (Ochsner, 2000; Schaefer and Philippot, 2005; Weymar et al., 2009; Schaefer et al., 2011). Event-related potential (ERP) studies are increasingly being used to examine the neural mechanisms of emotional memory. Findings from these studies have suggested that the effect of emotion on different memory retrieval processes are responsible for the unique features of emotional memory (Johansson et al., 2004; Inaba et al., 2005; Schaefer et al., 2009, 2011; Weymar et al., 2009).

Memory retrieval involves two separate processes. The first process, familiarity, occurs when people feel that someone or something is familiar, but they cannot recall any detailed information. Familiarity is thought to vary continuously. It involves the intense activation of an item in the memory, known as “memory strength,” which might produce internal signals that form the basis for feelings of familiarity (Gonsalves et al., 2005). The second process, recollection, refers to the ability of people to recall an item along with details of the encoding context (Rugg and Curran, 2007).

The old/new effect as measured by ERPs is a useful approach for examining recollection and familiarity. The key feature of the ERP old/new effect is that ERPs triggered by correctly classified “old” items are more positive than ERPs triggered by correctly classified “new” items in the test phase. Previous studies have revealed that the LPC old/new effect, which is a parietal maximum late positive component from 500–800 ms, reflects recollection process, and that the FN400 old/new effect, which is an earlier mid-frontal negative deflection from 300–500 ms, reflects familiarity process (Rugg et al., 1998; Rugg and Curran, 2007). Some researchers have suggested that FN400 is not the electrophysiological index of familiarity, but rather indicates conceptual priming (Paller et al., 2007; Hou et al., 2013). Familiarity and recollection are typically distinguished with the Remember-Know (R/K) paradigm or recognition confidence assessment. Specifically, remembered items (old items recalled with vivid detail) or high-confidence items index recollection, while known items (old items that cannot be recalled with detail) or low-confidence items index familiarity (Yonelinas, 2002).

To date, ERP studies of emotional memory have provided inconsistent findings regarding the effect of emotion on these two electrophysiological correlates of different memory processes. In some reports, emotional items significantly enhanced LPC amplitude (i.e., the index of recollection) compared to neutral items, but had no effect on FN400 (i.e., the index of familiarity; Johansson et al., 2004; Inaba et al., 2005; Schaefer et al., 2009, 2011; Weymar et al., 2009). However, in other studies, emotion enhanced the amplitudes of both LPC and FN400 compared to neutral material (Inaba et al., 2005; Schaefer et al., 2009, 2011).

One potential reason for these inconsistent findings may be that the studies did not separate the influences of valence (a continuum ranging from unpleasant to pleasant) and arousal (a continuum ranging from calm to excited). Valence and arousal have been correlated with different physiologically active modes (Bernat et al., 2006) and neural mechanisms (Kensinger and Schacter, 2006; Olofsson et al., 2008). Given that valence and arousal are different psychological processes, their effects on memory may be different. Valence and arousal have been shown to enhance emotional memory through different neural encoding processes, with memory enhancement of valenced or arousing information relying on conceptual encoding processes or sensory encoding process, respectively (Kensinger, 2009; Mickley Steinmetz and Kensinger, 2009).

An fMRI study found that compared to the successful encoding of unpleasant pictures, encoding of pleasant pictures is related to larger activation of the superior and middle frontal gyrus which are always activated by the conceptual and elaborate processing strategy (Mickley Steinmetz and Kensinger, 2009). In addition, enhancement of semantic integration in encoding can increase the likelihood of familiarity-based recognition memory (Meyer et al., 2007, 2010). Some neuroimaging studies found that the encoding of later-known pleasant information recruited the cingulate gyrus, bilateral frontal areas, and parietal area (regions associated with episodic and semantic retrieval and self-referential processing; see e.g., Northoff and Bermpohl, 2004; Northoff et al., 2006) more than the encoding of later-known unpleasant or neutral information (Mickley and Kensinger, 2008). These fMRI findings suggest that sematic encoding tendency of pleasant information may lead to increased feelings of familiarity; thus, we believe that due to the sematic encoding of pleasant stimuli, pleasant valence may enhance the retrieval process based on familiarity. Conversely, the encoding of highly arousing items recruited the inferior parietal lobe, the middle occipital gyrus, and the parahippocampal gyrus (Mickley Steinmetz and Kensinger, 2009), which are regions associated with sensory processing, visual attention (Mangun et al., 1998; Clower et al., 2001), and visual memory (Squire et al., 2004). These findings suggest that memories of arousing information involve increased sensory processing during encoding, which means that more sensory characteristics are attended and processed at encoding stage. Therefore, more details were attended and processed prior at the sensory level, especially those physical characteristics (e.g., shape, color) because of the sensory encoding tendency (Kensinger, 2009). Neuroimaging studies have demonstrated that processes recruited during retrieval can sometimes reflect the recapitulation of processes engaged during an encoding episode (e.g., Kahn et al., 2004; Kensinger, 2009). It makes sense that the information in which people orient toward at encoding would affect the types of information that would be retrieved (Kensinger, 2009). Thus, we believe that the sensory encoding tendency of high arousal stimuli may make the subjects recall more sensory details.

ERP technology can be used to test the above hypothesis. In addition to FN400 and LPC in memory retrieval phase, there are two ERP components in the encoding stage of emotional memory, which is considered to be the index of elaborative encoding and sensory encoding of emotional stimuli respectively (Palomba et al., 1997; Dolcos and Cabeza, 2002; Paller and Wagner, 2002; Koenig and Mecklinger, 2008; Olofsson et al., 2008). The former is a slow wave over the frontal sites which was evoked by pleasant stimuli (Koenig and Mecklinger, 2008), and the latter is a long-lasting positivity over the centro-parietal sites which was usually evoked by arousing stimuli (Palomba et al., 1997; Dolcos and Cabeza, 2002; Olofsson et al., 2008). If the semantic encoding of pleasant stimuli leads to the enhancement of familiarity, the positive image will trigger larger amplitude of the slow wave at frontal area in encoding phase, and a larger FN400 old/new effect in retrieval phase. If the sensory encoding of high arousal stimuli leads to the enhancement of recollection, the high arousal pictures will evoke a more positive potential over the centro-parietal sites in encoding stage, and a larger LPC old/new effect in retrieval stage.

A recent ERP study (Van Strien et al., 2009) used a continuous recognition procedure to investigate the effects of valence and arousal on the electrophysiological correlates of recognition memory at the retrieval stage. This study employed an orthogonal design to manipulate valence and arousal separately. The result showed that valence affected the amplitude of the old/new effect at frontal sites in an early time window (200–300 ms and 300–400 ms), whereas arousal affected the amplitude at parietal sites in a later time window (750–1000 ms).

In the continuous recognition procedure, items are presented twice in one block with intervening trials between the first and second presentation. Subjects are asked to decide whether the item was presented before. So the continuous recognition procedure involves alternating encoding and retrieval and the lag between the first and second presentation of an item is considerably shorter than with the study-test procedure. Furthermore, the “familiarity vs. recollection” distinction may be less applicable to the early mid-frontal and later parietal old/new effects in this research (Van Strien et al., 2009). The previous study suggested that, in a continuous recognition paradigm, the early mid-frontal old/new effect may reflect implicit memory, while the later parietal old/new effect may reflect the strength of the memory trace (Van Strien et al., 2005, 2007). In contrast, in a study-test procedure, the subjects were asked to study the items first, after a few minutes, the memory of these items were tested. This procedure separates encoding and retrieval stages and enables us to investigate the meaning of the early mid-frontal and the later parietal old/new effects (i.e., corresponding to familiarity and recollection, respectively; Rugg et al., 1998; Rugg and Curran, 2007). Therefore, we employed the study-test procedure, in conjunction with the recognition confidence assessment and an orthogonal design to investigate the impact of valence and arousal on familiarity and recollection.

Furthermore, there are some studies found an interaction effect of valence and arousal on memory encoding (Kensinger and Corkin, 2004; Mickley Steinmetz et al., 2010; Feng et al., 2012, 2014). A behavior study found that the memory enhancement for positive high arousal words relied on control encoding processes, and the negative high arousal word was encoded automatically (Kensinger and Corkin, 2004). An fMRI study found that high arousal negative stimuli enhanced the connection between amygdala and middle occipital gyrus, while high arousal positive stimuli reduced this connection (Mickley Steinmetz et al., 2010). ERPs studies found that negative pictures evoked a larger amplitude of P2, N2 and LPP components than positive pictures at the high-arousal level, whereas the reverse was true at the low-arousal level (Feng et al., 2012, 2014). These results indicate that there are interactive effects of valence and arousal on encoding of emotional stimuli. Thus the effect of valence and arousal on retrieval process would be more comprehensive which deserved to investigated in the present study.

The present research sought to determine the relationship between two dimensions of emotion (valence and arousal) and two memory processes (familiarity and recollection) by employing the orthogonal design to manipulate valence and arousal separately. The mechanism of how the two emotional dimensions affect the two memory processes was also investigated. The study-test paradigm was utilized to separate the encoding and retrieval stages. We anticipated that valence would affect FN400 old/new effect which indexed familiarity, whereas arousal would affect LPC old/new effect which indexed recollection. These effects may be due to the conceptual encoding tendency of pleasant stimuli and the sensory encoding tendency of high arousal stimuli.

## Materials and Methods

### Participants

Thirty healthy right-handed students (17 males, 13 females) participated in the experiment. The mean age of the participants was 21.70 (*SD* = 2.65), all of them had normal or corrected-to-normal vision. Three participants with less than 16 artifact-free trials in at least one relevant condition were excluded from the analysis, leaving a final sample of twenty-seven participants (15 males, 12 females, mean age of 21.70, *SD* = 2.66). All subjects signed an informed consent and got paid for their participation. This research was approved by the Human Research Ethics Committee at Capital Normal University.

### Stimuli and Design

We chose 432 pictures from the International Affective Picture System (IAPS; Lang et al., 1999), according to their normative scores on the valence and arousal dimensions, and applied the orthogonal design to those pictures. Specifically, arousal levels can be matched in valence ratings, while unmatched in arousal ratings; and different valence levels can be matched in arousal ratings, while unmatched in valence ratings. The pictures were divided into five subsets: (1) unpleasant low-arousal; (2) unpleasant high-arousal; (3) pleasant low-arousal; (4) pleasant high-arousal; and (5) neutral stimuli. There were 72 pictures in each of the four emotional sets and 144 pictures in the neutral set. The values of valence and arousal in each subset are displayed in Table 1.

**Table 1:** **The mean ratings of valence and arousal in all emotional subsets (with standard errors of the mean in parentheses)**.

	IAPS rating				Report rating			
	Low arousal		High arousal		Low arousal		High arousal	
	Valence value	Arousal value	Valence value	Arousal value	Valence value	Arousal value	Valence value	Arousal value
Unpleasant	2.93 (0.06)	4.72 (0.06)	2.86 (0.08)	6.05 (0.04)	2.25 (0.07)	5.35 (0.24)	2.28 (0.07)	6.86 (0.22)
Pleasant	7.34 (0.05)	4.62 (0.05)	7.36 (0.04)	5.93 (0.07)	7.40 (0.07)	5.11 (0.23)	7.43 (0.07)	6.42 (0.24)

As an individual’s emotional experience is based on one’s cognitive appraisal (Lazarus, 1991), the same stimulus may evoke different feelings for different people. For example, a picture of a dog may make subject A feel happy, but not affect subject B. Thus, this picture would be a pleasant stimulus for subject A and a neutral stimulus for subject B. Distributing emotional pictures to different categories according to an individual’s experience allows for a more exacting investigation of emotion.

To ensure that emotion was effectively elicited, the orthogonal design was applied twice. A second-pass orthogonal design was employed during data analysis to match valence and arousal according to participants’ verbal reports. First, subjects rated the valence and arousal of the IAPS pictures on two 9-point scales according to their feelings. Then, the emotional pictures were redistributed according to these ratings. Pictures with valence scores of 1–3 were assigned to the “unpleasant” group; those with valence scores of 4–6 were assigned to the “neutral” group; and those with valence scores of 7–9 were assigned to the “pleasant” group. Unpleasant and pleasant pictures were sorted in ascending order according to the subject’s reported arousal ratings; the half with lower or higher arousal ratings was assigned to the “low-arousal” or “high-arousal” level, respectively, in both valence sets (except neutral stimuli). Finally, we identified the five subsets defined above (unpleasant low-arousal, unpleasant high-arousal, pleasant low-arousal, pleasant high-arousal, and neutral stimuli), and reassigned the emotional stimuli to each emotional subset. We refer to this design, wherein emotional pictures were distributed into different conditions according to an individual’s subjective experience, as the “subject-oriented orthogonal design”.

The subjects reported values of valence and arousal in each subset are also displayed in Table 1. Result of paired-*t* test on normative ratings and subjects’ ratings showed the same pattern. The arousal levels of unpleasant and pleasant pictures were comparable (norm: 5.38 vs. 5.28, *p* = 0.29; subject: 6.11 vs. 5.76, *p* = 0.111), low- and high-arousal pictures had comparable valence level (norm: 5.12 vs. 5.12, *p* = 0.99; subject: 4.82 vs. 4.85, *p* = 0.088). In addition, because neutral pictures were different from pictures in the other emotional subsets on valence (all *p*’s < 0.001) and arousal (all *p*’s < 0.001), we were unable to examine the effects of valence and arousal on memory in an independent fashion. Thus, neutral pictures were excluded from data analysis.

The entire picture set was separated into two equal sets (A and B) that were alternatively presented as “old” (previously studied) or “new” (unstudied) during the test stage. Picture presentation was counterbalanced across participants. Valence (*t* < 1.7; *p* > 0.10) and arousal (*t* < 1.5; *p* > 0.10) were not different between the A and B emotional subsets. Because participants only assessed the valence and arousal of studied pictures, the ratings of unstudied pictures were unknown. For this reason, we could not assign unstudied pictures to different subject-oriented emotional conditions for further analysis.

To assign new pictures to different subject-oriented conditions, we used the “AB matching list”. In this list, for each item in set A, there was a corresponding picture in set B that most matched the picture in set A in terms of valence and arousal (according to the IAPS normative ratings). For each subject, an unstudied item (e.g., from set A) was allocated to the same subject-oriented condition as the corresponding studied picture (e.g., from set B) determined by the AB matching list. Pictures with a maximum visual angle of 6.04° × 7.25° were presented within a gray box (subtending 7.5° × 7.5°) at a viewing distance of 1 m.

**Figure 1:**
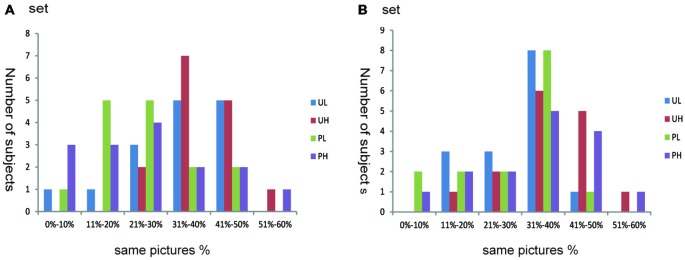
**Individual differences in emotion categorization**. Abscissa depicts the proportion of images that were divided into the same emotional set according to subjective appraisal and IAPS normative scores (i.e., the number of images that were divided into the same emotional set according to subjective appraisal and IAPS normative score divided by the number of images that divided into this emotional set according to the IAPS normative score alone). Ordinate depicts the number of subjects. Because participants only evaluated half of all pictures (set **A** or **B**), we calculated proportions according to the data of either (**A** or **B**), depending on which set the participants assessed. UL, unpleasant low-arousal; UH, unpleasant high-arousal; PL, pleasant low-arousal; PH, pleasant high-arousal.

To test the effectiveness of the subject-oriented orthogonal design, we calculated the proportion of images that were allocated into the same emotional sets according to subjective appraisal and IAPS normative scores. This calculation also accounted for the number of subjects in each proportion. For most participants (Figure 1), the proportion ranged from 30% to 50% (maximum ≤ 60%). Thus, if we used the emotional subset divided according to IAPS normative scores, approximately half of the emotional pictures were not effective at inducing an emotional response for most subjects.

### Procedure

The experiment was separated into a study stage and a test stage, separated by a 10-min interval, during which subjects played a video game (Tetris) on the computer. During the study stage, participants viewed pictures from one of the sets (A or B, counterbalanced across participants) displayed on a 17-inch screen. Pictures were presented pseudorandomly in four blocks of 54 scenes, with no more than three pictures from the same emotional subset presented consecutively. In each trial, a picture was presented for 2500 ms. Thereafter, participants were asked to rate the image using two successive 9-point Likert scales, assessing valence (unpleasant to pleasant) and arousal (calm to exciting), respectively. Participants indicated their emotional response on the Likert scale with a key press. To prevent primacy and recency effects, the first and last two items of each study block were filler items drawn from a remainder of IAPS pictures.

During the test stage, participants were given a surprise memory test in which they viewed all of the pictures (sets A and B), with half “old” items and half “new” items intermixed. Pictures were presented pseudorandomly in four blocks of 108 scenes, with no more than three consecutive “old” or “new” pictures, and no more than three consecutive similarly emotional pictures. In each trial, a fixation point was presented (a white cross on a black background) for a randomly varying duration (1000–1500 ms). A test picture was then displayed for 1000 ms, followed by presentation of a black screen for 1000 ms. Participants were asked to perform an old/new judgment and indicate their response with a keyboard press within these 2000 ms. After each recognition response, participants rated how confident they were in their recognition judgment using a 9-point Likert scale (for “new” judgments: 1 = not confident; 9 = absolutely confident; for “old” judgments: 1 = not confident; 8 = absolutely confident, but cannot recall any details, 9 = absolutely confident and can recall details).

Because vivid details are recalled along with the retrieved items during the recollection process, participants were instructed to make their highest confidence judgments only when they were absolutely sure that they saw an item and could recall details. Old items receiving confidence ratings of 9 were classified as high-confidence (remembering items). All remaining old items were assigned to the low-confidence category (knowing items; Wixted and Stretch, 2004; Weymar et al., 2009).

### ERP Recording

Electroencephalogram (EEG) was recorded continuously from 62 scalp sites with a NeuroScan SynAmps system (NeuroScan Inc. sterling, Virginia, USA) sampled at a rate of 500 Hz. Every electrode on the cap was carefully checked before the experiment to make sure they functioned well during the EEG recording. Vertical and horizontal electrooculograms (EOG) were recoded with two pairs of electrodes. One pair was placed above and below the left eye (VEOG) and the other pair was placed at the outer canthi of both eyes (HEOG). All scalp electrodes were referenced to the left mastoid during recording and rereferenced to the average of the right and left mastoid off line. The EEGs were recorded with a band pass of 0.05–100 Hz online, and filtered with a band pass of 0.05–40 Hz offline. Electrode impedances were kept below 5 kΩ. Eye movement artifacts were corrected by the method nested in Neuroscan (Semlitsch et al., 1986).Trials with a voltage exceeding ±75 μV at any electrode were excluded from analysis as artifacts. EEG data were segmented into epochs between 200 ms before and 1000 ms after picture onset. ERP waveforms were created through averaging EEG data for correct responses to old and new items separately for four emotional categories, resulting in at least 16 artifact-free trials in each condition per participant.

### Statistical Analysis

All dependent variables were subjected to repeated-measures analyses of variance (ANOVA). In all ANOVAs, degrees of freedom are adjusted with the Greenhouse–Geisser correction for nonsphericity. All the analyses conducted on the behavior and ERPs data were based on subjects’ ratings.

#### Behavior Data

A valence (unpleasant vs. pleasant) × arousal (low arousal vs. high arousal) repeated-measures ANOVA was conducted on the recognition performance of the old items with low confidence(LC) and high confidence(HC) judgments. Four indices was computed to measure the recognition performance including hit rate, false alarm, memory discrimination accuracy [Pr: P (hits) – P (false alarms)] and response bias scores [Br: P(false alarm)/P(1–Pr)] (Snodgrass and Corwin, 1988).

The confidence assessment which used in current study is similar to a modified R/K paradigm (Hou et al., 2013). Specifically, the old items with high confidence rating can be recalled along with vivid details, just like the remembered items in R/K paradigm, while the old items with low confidence from 1–8 cannot be recalled with any details, just like the know items in R/K paradigm. In order to obtain independent estimates of high and low confidence recognition, we refer the correction of K scores in R/K paradigm to correct the low confidence scores in current study (Yonelinas and Jacoby, 1995; Schaefer et al., 2011). The low confidence scores (i.e., the probability of an old item to receive a low confidence judgment) were corrected by dividing it by the probability of an old item to not receive a high confidence judgment (1−HC).

#### ERPs Data

At the encoding stage, we chose two ERPs indices occurred at frontal areas (F1, FZ, F2) between 350–800 ms and centro-parietal sites(CP1, CPZ, CP2) between 500–800 ms, which were supposed to index conceptual encoding and sensory encoding. A valence (unpleasant vs. pleasant) or arousal (low arousal vs. high arousal) one way repeated-measures ANOVA was conducted on these two ERPs to examine the effect of valence and arousal on different processing style.

The analysis of ERP data at the retrieval stage were conducted in two steps.

Step1: The topography and temporal dynamics of the ERP old/new effect were examined. In line with previous literature (e.g., Van Strien et al., 2009), nine regional averages were computed for each time window and each condition: left anterior (F3, F5, F7, FC3, FC5, FC7), midline anterior (F1, FZ, F2, FC1, FCZ, FC2), right anterior (F4, F6, F8, FC4, FC6, FC8), left central (C3, C5, C7, CP3, CP5, CP7), midline central (C1, CZ, C2, CP1, CPZ, CP2), right central (C4, C6, C8, CP4, CP6, CP8), left posterior (P3, P5, P7, PO3, PO5, PO7), midline posterior (P1, PZ, P2, POZ), and right posterior (P4, P6, P8, PO4, PO6, PO8). These regions spanning over anterior, central and posterior regions and left, midline and right laterality which constituted two independent variables: caudality (anterior, central, posterior) and laterality (left, midline, right). Based on a careful examination of our waveforms and a review of previous finding (Ally and Budson, 2007; Voss and Paller, 2007; Koenig and Mecklinger, 2008; Van Strien et al., 2009), we selected three time windows: 350–500, 500–800, 800–1000 ms. First, a four-way (old/new × caudality × laterality × epoch) repeated-measures ANOVA was conducted on regional averages of amplitude. Then a repeated-measures variance analysis in each time window was conducted to test the interaction of old/new and regions. At last, we analyzed the old/new effect in each region to ascertain the main region which old/new effect occurred.

Step2: In the brain area where old/new effects occurred, the influences of valence and arousal on the old/new effects were tested.

## Results

### Behavior Data

An overview of participants’ recognition memory performance is given in Tables 2, 3.

**Table 2:** **Probabilities of hit and false alarm of old items and measures of old-new discrimination (Pr) and Response Bias (Br) in low confidence condition (with standard errors of the mean in parentheses)**.

	Low arousal				High arousal			
	HIT	FA	Pr	Br	HIT	FA	Pr	Br
Unpleasant	0.57 (0.04)	0.17 (0.02)	0.40 (0.04)	0.33 (0.04)	0.55 (0.04)	0.18 (0.03)	0.35 (0.05)	0.32 (0.04)
Pleasant	0.58 (0.05)	0.19 (0.02)	0.38 (0.05)	0.38 (0.05)	0.70 (0.04)	0.18 (0.02)	0.51 (0.05)	0.50 (0.06)

**Table 3:** **Probabilities of hit and false alarm of old Items and measures of old-new discrimination (Pr) and Response bias (Br) in High Confidence Condition (with standard errors of the mean in parentheses)**.

	Low arousal				High arousal			
	HIT	FA	Pr	Br	HIT	FA	Pr	Br
Unpleasant	0.58 (0.04)	0.03 (0.01)	0.55 (0.04)	0.09 (0.03)	0.63 (0.05)	0.03 (0.01)	0.60 (0.04)	0.09 (0.03)
Pleasant	0.58 (0.04)	0.02 (0.01)	0.57 (0.04)	0.05 (0.01)	0.67 (0.04)	0.05 (0.01)	0.62 (0.05)	0.13 (0.03)

#### Hit Rate and False Alarm

The two-way repeated measures ANOVA of valence (pleasant vs. unpleasant) by arousal (low arousal vs. high arousal) yielded a main effect of valence [*F*_(1, 25)_ = 7.11, *p* = 0.013] and significant interaction among valence and arousal [*F*_(1, 25)_ = 5.85, *p* = 0.023] on hit rate of LC old items. Planned paired-*t* test showed that the hit rate of unpleasant and pleasant items in low arousal condition was comparable [0. 57 vs. 0.60, *t*_(28)_ = −0.43, *p* = 0.669]. The results also showed a main effect of arousal on hit rate of HC old items, the high arousal items were hit more accurate than low arousal ones [*F*_(1, 29)_ = 11.86, *p* = 0.002].

The repeated-measures ANOVA conducted on false alarm only found a significant interaction among valence and arousal on HC old items [*F*_(1, 29)_ = 6.47, *p* = 0.017]. Planned paired-*t* test showed that false alarm was higher for high arousal pictures than that for low arousal pictures in pleasant condition [0.02 vs. 0.05, *t*_(29)_ = −2.80, *p* = 0.009], but the false alarm of high arousal and low arousal pictures in unpleasant condition were similar [0.03 vs. 0.03, *t*_(29)_ = 0.27, *p* = 0.79].

#### Discrimination Accuracy and Response Bias

The valence by arousal repeated measures ANOVA yielded a main effect of valence [*F*_(1, 25)_ = 4.36, *p* = 0.047] and significant interaction among valence and arousal [*F*_(1, 25)_ = 8.59, *p* = 0.007] on Pr scores of LC old items. Planned paired-*t* test showed that the Pr scores of pleasant items was significant higher than those of unpleasant ones [0.34 vs. 0.52, *t*_(25)_ = 3.70, *p* = 0.001] in high arousal condition, but the Pr scores of unpleasant and pleasant items in low arousal condition were comparable [0.40 vs. 0.39, *t*_(28)_ = 0.71, *p* = 0.944]. The results also showed a main effect of arousal on Pr of HC old items, the high arousal items were discriminated more accurate than low arousal ones [*F*_(1, 29)_ = 5.63, *p* = 0.024]. For each emotional subsets, accuracy was reliably greater than 0 in both low and high confidence conditions[low confidence: all *t*’s > 7.31, *p*’s < 0.001; low confidence: all *t*’s > 12.91, *p*’s < 0.001], indicating that memory was above chance levels.

The analysis of response bias measures (Br) revealed a main effect of valence on low confidence levels [*F*_(1, 25)_ = 9.91, *p* = 0.004] with more liberal response bias for pleasant items than that for unpleasant ones. The analysis also found a marginal significant main effect of arousal [*F*_(1, 29)_ = 4.15, *p* = 0.051] and a significant interaction among valence and arousal on Br of HC old items [*F*_(1, 29)_ = 5.34, *p* = 0.028]. Planned paired-*t* test showed that high arousal pictures were associated with a more liberal response bias than low arousal pictures in pleasant condition [0.05 vs. 0.13, *t*_(29)_ = −2.81, *p* = 0.009], while in unpleasant condition, the response bias of high arousal pictures and low arousal pictures were comparable [0.09 vs. 0.10, *t*_(29)_ = 0.27, *p* = 0.79].

### ERP Data

#### Old/New Effect^1^

**Figure 2:**
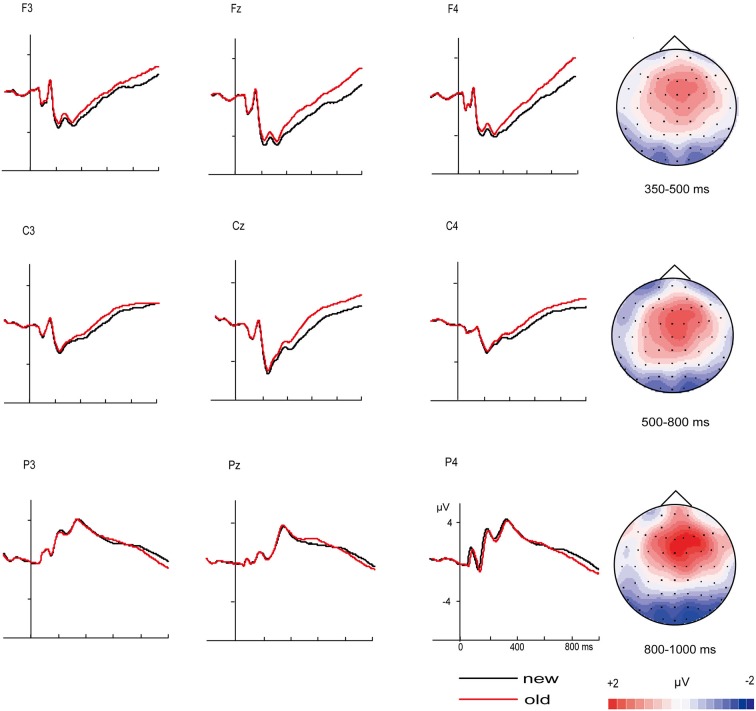
**ERP correlates and topographic map of old/new effect**. Grand-average event-related potentials (ERPs) from selected electrodes for “new” pictures vs. “old” pictures and topographic maps for the old/new effect (“old” minus “new” pictures).

Figure 2 displays the grand average ERPs old/new effect at selected electrodes for “new” vs. “old” pictures and the topographic distributions of the main old/new effects for all four time windows. As expected, correctly recognized old pictures elicited more positive-going ERPs than correctly rejected new pictures from 350 ms onward. Except main old/new effect, the ANOVAs also yielded significant interactions of old/new, caudality and laterality for the 350–500, 500–800 and 800–1000 ms time windows (Table 4). These interactions reflect the topographic distributions of the old/new effects. It can be seen from Figure 2 that the old/new effect was largest in the 500–800 ms time window, the early old/new effect (350–500 ms) was maximum at mid-frontal sites and the late old/new effect (500–800 ms) distributed widely with a rightward midline central maximum. In the 800–1000 ms time windows, the topographic voltage map indicates that the old/new effect mainly distributed at right anterior sites. A significant inverse old/new effect was observed at posterior in each time window.

**Table 4:** **ANOVA main and interaction effect of old/new in each epoch**.

		350–500 ms		500–800 ms		800–1000 ms	
	df	*F*	*P*	*F*	*P*	*F*	*P*
Old/new	1,29	44.48	<0.001	67.88	<0.001	11.41	0.002
Old/new × caudality	2,58	24.97	<0.001	48.91	<0.001	29.23	<0.001
Old/new × laterality	2,58	18.22	<0.001	8.78	0.001	5.76	0.014
Old/new × caudality × laterality	4,116	0.59	*ns*	6.58	0.001	2.96	*ns*

In order to test whether the effect of old/new effect was same in each electrode in the above brain areas (mid-frontal, right-frontal, midline central), mean ERP amplitudes of each electrode in these brain areas were analyzed with an ANOVA including the factors Electrode (6) and old/new (2). The results showed that besides the interactions of electrode × old/new during 800–1000 ms at right frontal [*F*_(5, 145)_ = 5.27, *p* = 0.002], the other interactive effects were not significant.

FN400 is always found at frontal sites, in this study similar effects were discovered in the 350–500 time window. As the ANOVA did not find a significant electrode × old/new interaction [*F*_(5, 145)_ = 0.47, *p* = 0.75], we chose three electrodes(F1, Fz, F2) to constitute the mid-frontal area, ANOVA based on the mean amplitudes recorded over the mid-frontal cluster in the 350–500 ms time window yielded a main effect of old/new [*F*_(1, 29)_ = 62.50, *p* < 0.001].

LPC is always found at parietal sites in previous literature between 500–800 ms, we also found a significant old/new effect at midline of central and central parietal sites during 500–800 ms [*F*_(1, 29)_ = 52.45, *p* < 0.001].

Literature review reveals that old/new effect of 800–1000 ms might index post-retrieval processing that usually occurs at right-frontal sites. Although there was an electrode × old/new interaction during 800–1000 ms at right frontal cluster [*F*_(5, 145)_ = 5.27, *p* = 0.002], *post hoc* analyses indicated that the amplitude of old items were more positive than new items at every electrode (all *p*’s < 0.05). We chose three electrodes (F4, F6, F8) to constitute the right-frontal area, ANOVA based on the mean amplitudes recorded over the right-frontal cluster in the 800–1000 ms time window yielded a main effect of old/new [*F*_(1, 29)_ = 23.14, *p* < 0.001].

#### Interaction Effects of Valance, Arousal and Old/New

As the significant old/new effects were found at mid-frontal sites (350–500 ms), midline central sites (500–800 ms), and right frontal sites (800–1000 ms), valence (or arousal) × old/new × electrode three way repeated measures ANOVAs were conducted in these areas and time windows to discover the influence of valence and arousal on old/new effects.

**Figure 3:**
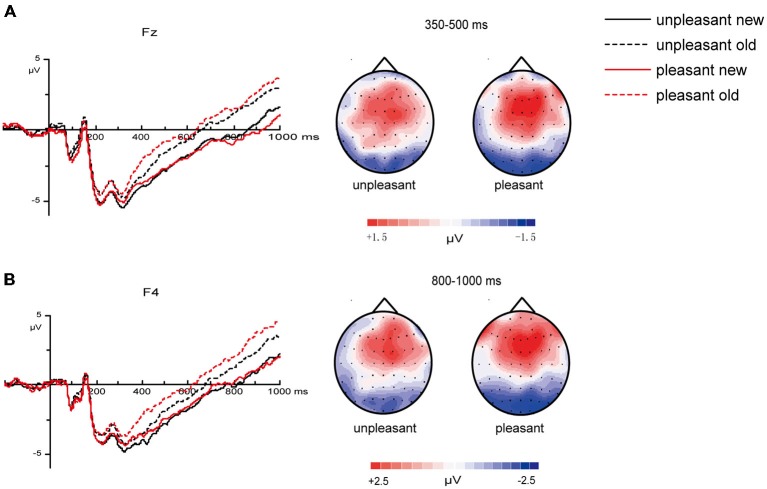
**Influences of valences on 350–500, 800–1000 ms old/new effects**. **(A)** Grand average ERPs of unpleasant “new” vs. “old ” pictures and pleasant “new” vs. “old ” pictures at mid-frontal sites and topographic maps for the old/new effect in different valence levels between 350–500 ms (“old” minus “new” pictures). **(B)** Grand average ERPs of unpleasant “new” vs. “old ” pictures and pleasant “new” vs. “old ” pictures at right frontal sites and topographic maps for the old/new effect in different valence levels between 800–1000 ms (“old” minus “new” pictures).

**Figure 4:**
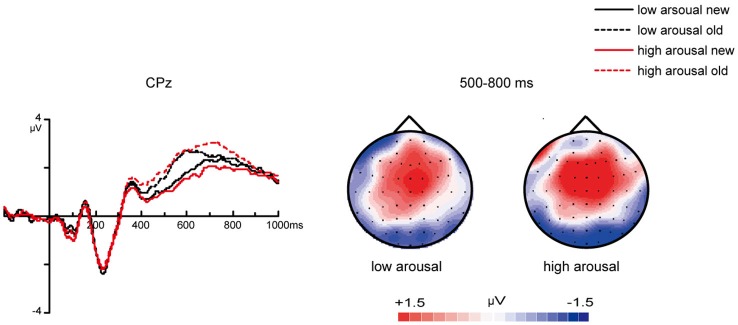
**Influences of arousal on 500–800 ms old/new effects**. Grand average ERPs elicited by low arousal “new” vs. “old” pictures and high arousal “new” vs. “old” pictures at midline central sites and topographic maps for the old/new effect in different arousal levels between 500–800 ms (“old” minus “new” pictures).

The analyses revealed a marginal significant interaction of valence and old/new [*F*_(1, 26)_ = 4.19, *p* = 0.051] between 350–500 ms at mid-frontal sites (Figure 3), a significant interaction of arousal and old/new [*F*_(1, 26)_ = 8.88, *p* = 0.006] between 500–800 ms at midline central sites (Figure 4) and a significant interaction of valence and old/new between 800–1000 ms at right frontal sites [*F*_(1, 26)_ = 6.37, *p* = 0.018; Figure 3]. All the ANOVAs above did not find interactions of valences/arousal × old/new × electrodes, which indicate that all electrodes have same effect in the same brain area.

**Figure 5:**
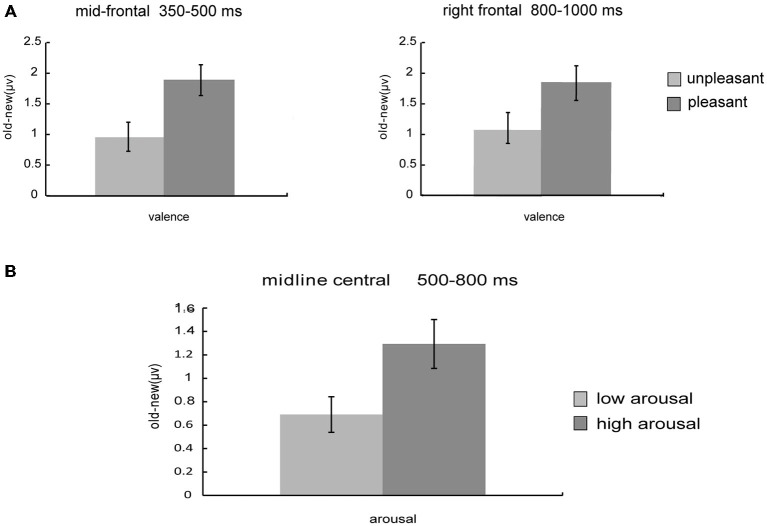
**Influences of valence and arousal on old/new effects. (A)** Old/new effect of unpleasant vs. pleasant pictures. ERPs used in the analyses are averaged across channels within the mid-frontal (350–500 ms) and right frontal (800–1000 ms) clusters. **(B)** Old/new effect of low arousal vs. high arousal pictures. ERPs used in the analyses are averaged across channels within the midline central (500–800 ms) clusters.

Planned paired-*t* test was applied to further compare the old/new effect (“old”–“new”) of different valence and arousal level (Figure 5). Results showed that during 350–500 ms, the old/new differences of pleasant pictures were larger than those of unpleasant pictures at mid-frontal sites [*t*_(26)_ = −2.05, *p* = 0.051]; in the 500–800 ms time window, the old/new differences of high-arousal pictures were larger than those of low-arousal pictures [*t*_(26)_ = −2.98, *p* = 0.006]; and for the 800–1000 ms time window, the old/new differences of pleasant pictures were larger than those of unpleasant pictures at right frontal sites [*t*_(26)_ = −2.52, *p* = 0.018].

#### The Effect of Valence and Arousal at Encoding Stage

**Figure 6:**
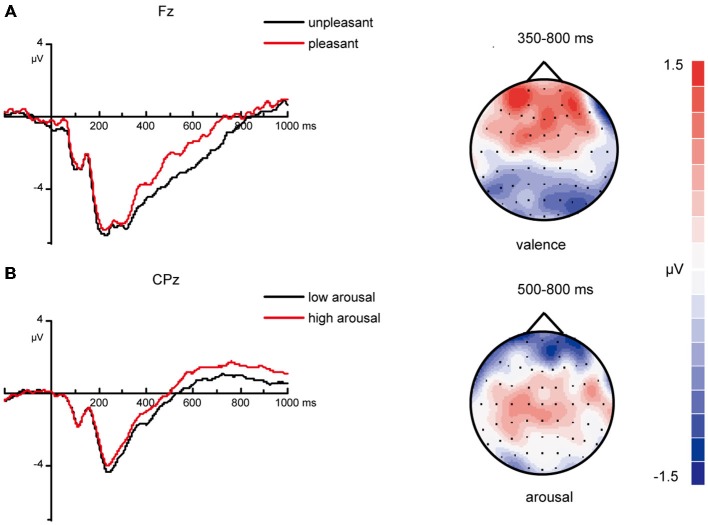
**ERP correlates and topographic map of different valenced and arousaled pictures in encoding stage**. **(A)** Grand average ERPs of unpleasant vs. pleasant pictures in encoding stage at mid-frontal areas and topographic maps of “pleasant” – “unpleasant” difference from 350–800 ms. **(B)** Grand average ERPs of low arousal vs. high arousal pictures in encoding stage at midline central areas and topographic maps of “high-arousal”—“low-arousal” difference from 500–800 ms.

For ERPs from 350–800 ms at frontal areas, the main effect of valence was significant [*F*_(1, 26)_ = 10.19, *p* = 0.004]. Slow waves evoked by pleasant stimuli were more positive than those evoked by unpleasant stimuli (Figure 6). However, the effect of arousal was not significant [*F*_(1, 26)_ = 0.04, *p* = 0.85].

For ERPs from 500–800 ms at centro-parietal areas, the main effect of arousal was significant [*F*_(1, 26)_ = 11.10, *p* = 0.003]. The amplitudes evoked by high-arousal items were more positive than the amplitudes evoked by low-arousal items (Figure 6). The effect of valence was not significant [*F*_(1, 26)_ = 1.88, *p* = 0.18].

## Discussion

In the present study, we investigated the neural basis of the enhancement of emotional memory. We revealed different effects of valence and arousal on different subtype of old/new effect. Specifically, valence influenced the FN400 old/new effect and late positivity over right frontal, while arousal influenced the LPC old/new effect. These results suggested that valence affected familiarity, whereas, arousal affected recollection. And the results of encoding stage suggested that the effects at retrieval may be due to the conceptual encoding tendency of pleasant stimuli and the sensory encoding tendency of high arousal stimuli.

### Behavior Data

Behavioral data demonstrated a dissociable effect of valence and arousal on the accuracy of discriminating low- and high-confidence items. Valence or arousal affected the discrimination accuracy of low- or high-confidence items, respectively, while the effect of valence and arousal on hit rate showed a same pattern. These findings support our hypothesis that valence affects familiarity and arousal affects recollection.

Pleasant pictures were rated as more familiar than unpleasant pictures, an interaction was found between valence and arousal on the hit rate and Pr values of low-confidence items. This interaction suggests a more accurate discrimination of pleasant than of unpleasant items in the high-arousal condition, but the discriminations of pleasant and unpleasant items in the low-arousal condition are comparable. This interaction may reflect the relationship between pleasant experiences and familiarity-based recognition memory; specifically, the stronger the pleasant experience is, the better the familiarity-based recognition memory will be.

Another possible reason is that the individual tends to use conceptual process to encode pleasant high arousal stimuli, and use sensory process to encode unpleasant high arousal stimuli (Kensinger and Corkin, 2004; Mickley Steinmetz et al., 2010). However, for HC items, we did not find that the individuals recalled more details for unpleasant pictures than for pleasant pictures in the high-arousal condition. Thus, this explanation needs further investigation.

No interaction between the valence and arousal on the memory performance was found for high confidence items, the high-arousal pictures were remembered with more detail than low-arousal pictures whatever the valence is. Although some studies showed that the valence effect on neuro activity was opposite at high arousal condition compared to low arousal condition (Feng et al., 2012, 2014). The finding of current study can also be interpreted within existing framework regarding the role of the amygdala in emotional memory. Mass of studies found that the amygdala responded to arousing stimuli regardless of valence (Cahill, 2000; Canli et al., 2002; McGaugh, 2004). The connection between amygdala and wide posterior brain regions, including fusiform gyrus and the middle occipital gyrus, at the encoding and consolidation stage, improve the sensory process of high arousal stimuli, which result in better recognition performance of high arousal HC items in present study.

Pleasant pictures were associated with a more liberal response bias than unpleasant pictures for low-confidence items. Within high-confidence items, high-arousal pictures were associated with a more liberal response bias than low-arousal pictures in the pleasant condition. The analysis on the probability of false alarm showed a similar effect. In fact, the results of the false alarm rate and Br measures suggested a positive correlation between pleasant experiences and a liberal response bias. It is not clear why the participants responded to pleasant items with a more liberal bias. The “feelings-as-information” hypothesis suggests that good feelings signal a benign environment, in which individuals tend to employ heuristic processing. Heuristic processing causes rough processing of external information and more mistakes, because mistakes do not pose threats to the survival of the individual (Schwarz, 2002). This effect could contribute to a more liberal response bias of pleasant items.

### Event-Related Potentials

#### Conceptual Encoding Tendency Lead to the Impacts of Valence on FN400

Individuals retrieve emotional materials of different valences in different ways. Unpleasant events usually can be recalled, whereas neutral and pleasant stimuli are recognized only based on familiarity (Ochsner, 2000; Dolcos et al., 2004; Johansson et al., 2004; Kensinger and Corkin, 2004). Here, we found that the effect of valence on FN400, a familiarity index. Old/new differences are significantly larger for pleasant than for unpleasant stimuli. We also found a more positive amplitude of a frontal slow wave evoked by pleasant pictures relative to those of unpleasant ones. This result suggests that the pleasant items are mainly encoded by conceptual process and recognized by familiarity more than unpleasant items. The reason behind enhanced familiarity process for pleasant items may be the different encoding pattern of pleasant and unpleasant items, specifically, the encoding of pleasant items were more elaborate than unpleasant items.

Previous study found that pleasant emotion can lead to a broadening of attention and cognitive flexibility, suggesting that people with pleasant moods tend to process information holistically and conceptually (Fredrickson and Branigan, 2005; Rowe et al., 2007), but often miss details (Gasper and Clore, 2002). Neuroimaging data support this view. An fMRI study used an orthogonal design to study the neural mechanisms underlying the encoding of valence and arousal stimuli. Valence and arousal resulted in differences in subsequent memory performance. The authors proposed that pleasant emotions triggered an elaborate processing strategy, which led to frontal area activation. Compared to the successful encoding of unpleasant pictures, encoding of pleasant pictures is related to larger activation of the superior and middle frontal gyrus (Mickley Steinmetz and Kensinger, 2009), these brain regions are related to conceptual and elaborative processing (Reinders et al., 2003). An ERP study also observed a slow-wave activity in the frontal areas between 250–1000 ms which was more positive for pleasant than for unpleasant or neutral stimuli at encoding stage, paralleled by enhanced memory performance (Koenig and Mecklinger, 2008). In addition, the subsequent remembered items also elicited a more positive frontal slow wave than subsequent forgotten items in the encoding stage, which indicates that this frontal slow wave in encoding stage reflects the use of elaborate mnemonic strategies.

On the other hand, enhancement of semantic integration in encoding can increase the likelihood of familiarity-based recognition memory (Meyer et al., 2007, 2010). Another fMRI study found greater involvement of the cingulate gyrus, bilateral frontal, and parietal areas (brain regions are associated with episodic and semantic retrieval and self-referential processing; see, e.g., Northoff and Bermpohl, 2004; Northoff et al., 2006) in encoding later-known pleasant information compared to later-known unpleasant or neutral items (Mickley and Kensinger, 2008). In the present study, the frontal slow wave between 350–800 ms evoked by pleasant picture was also more positive than those evoked by unpleasant ones, paralleled by enhanced memory performance of LC old items and enhanced FN400 old/new effect. Based on previous study, we suggest that the conceptual and elaborative processing evoked by pleasant items results in stronger familiarity for these positive items.

#### Sensory Encoding Tendency Lead to the Impact of Arousal on LPC

We found that arousal had a significant effect on the recollection indicator, LPC. During the 650–800 ms epoch, high-arousal pictures evoked larger LPC old/new effects relative to low-arousal pictures. We also found the effect of arousal on centro-parietal positivity at encoding stage. The ERP amplitude evoked by high-arousal items was more positive than the amplitude evoked by low-arousal items. These results suggested that the encoding of high arousal items is more relied on sensory process compared to low arousal items and the retrieval of them is more based on recollection. The reason behind enhanced recollection process for high arousal items may be the sensory encoding tendency of high arousal items.

At the encoding stage, arousing information that was subsequently remembered was also linked with activity in wide posterior brain regions, including the inferior parietal lobe and middle occipital gyrus (Mickley Steinmetz and Kensinger, 2009). These brain regions are associated with sensory processing, visual attention (Mangun et al., 1998; Clower et al., 2001), and visual memory (Squire et al., 2004). Neuroimaging studies have demonstrated that processes recruited during retrieval can sometimes reflect the recapitulation of processes engaged during an encoding episode (e.g., Kahn et al., 2004; Kensinger, 2009). It makes sense that the information in which people orient toward at encoding would affect the types of information that would be retrieved (Kensinger, 2009). The effect of arousal on recollection may be due to the activation of posterior brain regions associated with sensory processing and visual attention during the encoding of arousing information. Increased activity in these structures may cause individuals to pay more attention to details during sensory processing of arousing events, such that more sensory details are recalled later (MacKay et al., 2004; Mather and Nesmith, 2008).

Similar results were also found in ERPs study. Numerous studies have reported a long-lasting elevation of ERP positivity in response to arousing pictures (for review, see Olofsson et al., 2008). Palomba et al. (1997) found that arousing unpleasant/pleasant stimuli, which were recalled more frequently than neutral or relatively low-arousal stimuli, elicited more positive-going slow waves in Pz and Cz that appeared within 300–900 ms during the encoding stage. Dolcos and Cabeza (2002) found a similar positive slow-wave arousal effect at the centro-parietal area from 400–600 ms of the encoding stage. This affective slow wave was sensitive to subsequent memory in a detail recall test; relative to neutral pictures, arousing pictures amplified the slow wave, and the increased amplitude corresponded to enhanced recognition memory performance. These findings have been postulated to reflect the enhanced encoding of arousing stimuli.

The centro-parietal slow wave between 500–800 ms was also found in the present study. Just as previous studies, the potential was more positive for high arousal pictures, paralleled by enhanced memory performance of HC old items and enhanced LPC old/new effect. In combination with the results of others, we believe that this positive slow wave reflects stronger encoding activities for high-arousal stimuli, which led to more sensory details were encoded. This heightened effect could lead to more accurate discrimination of high-confidence old items and more positive LPC amplitudes for high-arousal than for low-arousal pictures. Thus, arousal appears to enhance the recollection process during the retrieval stage.

#### Effects of Valence on Post-Retrieval Processing Components

We observed a positive right-frontal slow wave from 800–1000 ms. Late ERP effects in the right-frontal area may be related to post-retrieval verification and monitoring, especially when features or details of the memory require evaluation (Allan et al., 1998). Valence affected this component; specifically, old/new differences for pleasant pictures were significantly greater than for unpleasant pictures.

The effect of valence on this late slow-wave component may reflect the difficulty in processing pleasant information, especially when the pleasant information needs to be recalled with detail. When the memory task is difficult (e.g., detailed recall of information), additional processing is needed for successful recollection. This processing may be reflected by a larger activation of parietal or frontal areas in later epochs (Ally and Budson, 2007). It is postulated that parietal activity involves the actual matching of stored representations with perceptual representations (Addis and McAndrews, 2006; Ally and Budson, 2007), whereas frontal activity involves executive processing that helps direct memory retrieval search attempts (Baddeley, 1995). Because people tend to process pleasant stimuli holistically and conceptually (Fredrickson and Branigan, 2005; Rowe et al., 2007), it is possible that there are few representations of local or specific information for pleasant stimuli. Therefore, retrieval details of pleasant items is more difficult and requires greater executive processing, reflected by a more positive frontal slow wave from 800–1000 ms.

Since the “subject-oriented orthogonal design” was used in the present study, there were only nine subjects whose artifact-free trials were more than 16 in the four emotional subsets (UL, UH, PL, PH). Thus, we cannot investigate the interaction of valence and arousal in the present study. The results of the behavioral data implied that the FN400 old new effect of high arousal pleasant pictures may be larger than that of low arousal pleasant pictures. Post retrieval process of high arousal pleasant items should be stronger than that of low arousal pleasant items if the enhancement of familiarity retrieval process, which occurred on the pleasant stimuli, is due to the enhancement of conceptual code tendency of pleasant items. However, the valence by arousal effect should not be found at the LPC old new effect according to the results of behavior data. Future studies should be done to test the validity of these inferences.

In summary, the dissociable effects of valence and arousal on different subtypes of old/new effect which may index different memory processes may be due to the different processing strategies for valence and arousal information. Based on current and previous findings, we believe that pleasant information is processed using a conceptual and elaborative processing strategy, which affects familiarity and the post-retrieval process. In contrast, high-arousal information tends to be processed via enhanced sensory processing, leading individuals to pay more attention to details in the sensory processing stage.

## Conflict of Interest Statement

The authors declare that the research was conducted in the absence of any commercial or financial relationships that could be construed as a potential conflict of interest.

## Footnotes

^1^The analysis of the old/new effect was conducted on the data collapsed across all five emotion conditions (i.e., including the neutral condition).
