# Corrigendum: Dissociable Effects of Valence and Arousal on Different Subtypes of Old/New Effect: Evidence from Event-Related Potentials

**DOI:** 10.3389/fnhum.2016.00098

**Published:** 2016-03-10

**Authors:** Huifang Xu, Qin Zhang, Bingbing Li, Chunyan Guo

**Affiliations:** ^1^Beijing Key Laboratory of Learning and Cognition, Department of Psychology, Capital Normal UniversityBeijing, China; ^2^Office of Degree, Graduate School of Beijing, University of Chinese MedicineBeijing, China

**Keywords:** valence, arousal, familiarity, recollection, post-retrieval processing, conceptual, sensory

The information of “affiliation 1” of the authors in the online version of our manuscript is incorrect. It should be “Beijing Key Laboratory of Learning and Cognition, Department of Psychology, College of Education, Capital Normal University, Beijing, China.”

## Author contributions

HX: Substantial contributions to the conception or design of the work; the acquisition, analysis, interpretation of data for the work; drafting the work or revising it critically for important intellectual content; and agreement to be accountable for all aspects of the work in ensuring that questions related to the accuracy or integrity of any part of the work are appropriately investigated and resolved. QZ, BL: Drafting the work or revising it critically for important intellectual content and agreement to be accountable for all aspects of the work in ensuring that questions related to the accuracy or integrity of any part of the work are appropriately investigated and resolved. CG, Drafting the work or revising it critically for important intellectual content; agreement to be accountable for all aspects of the work in ensuring that questions related to the accuracy or integrity of any part of the work are appropriately investigated and resolved; and final approval of the version to be published.

### Conflict of interest statement

The authors declare that the research was conducted in the absence of any commercial or financial relationships that could be construed as a potential conflict of interest.

